# Prevalence and determinants of self-medication among medical undergraduates: a cross-sectional study

**DOI:** 10.1097/MS9.0000000000004450

**Published:** 2025-12-05

**Authors:** Ehtisham Rehman, Zain Ul Abideen, Hiba Ashfaq, Saba Riaz, Hifza Arshad, Neelab Jamil, Sana Javed, Irfan Ullah, Sardar Noman Qayyum, Ahmad Mohammad Saifan

**Affiliations:** a Department of Internal Medicine, Bacha Khan Medical College, Mardan, Pakistan; b Department of Internal Medicine, Lebanese University Faculty of Medical Sciences, Beirut, Lebanon

## Abstract

**Introduction:**

Self-medication is the practice of accessing and using different drugs for various purposes, including self-diagnosis and self-treatment without consulting a healthcare professional. Health science students, particularly medical students, are at the forefront of the future healthcare workforce. This KAP study provides insights into the prevalence, attitude, knowledge, and practices of self-medication among medical students.

**Methods:**

The study employed an institutional-based cross-sectional study design and was conducted from May 2024 to August 2024. The study had a sample size of 203, calculated through openepi.com with a 95% confidence interval and a 5% margin of error. The participants of the study were selected randomly using a two-stage stratified random sampling technique.

A pre-tested questionnaire, adapted from a similar study, was compiled in English, and all vague and unclear questions were rephrased or removed from the questionnaire. All the data collectors were trained, and data quality was ensured through supervision by the research team. The ethical aspects were upheld by obtaining informed consent, ensuring voluntary participation, maintaining participant confidentiality, and obtaining approval from the ethics review board.

**Results:**

191 students (48 from first year, 47 from second year, 48 from third year, 22 from fourth year, and 26 from final year) responded to the questionnaire, with a response rate of 94%. 76.9% of the respondents had practiced self-medication at least once within the last 6 months. All the students in the study had satisfactory knowledge about self-medication, with a mean knowledge score of 5.32 ± 0.86. 69.6% had a positive attitude towards self-medication, with a mean attitude score of 21.26 ± 3.12. The most commonly self-consumed drugs were analgesics (28.3%), followed by self-consumption of more than one drug (26.2%) during the last 6 months. 33.5% of the participants self-treated for more than one indication, followed by 28.3% for headache and 12.0% for fever during the last 6 months.

**Conclusion:**

The study findings indicate that there is a significantly high prevalence of self-medication practice among medical students. A majority of the students had good knowledge and a positive attitude toward self-medication, suggesting a high level of awareness and understanding regarding self-medication. Most students prefer self-medication because it is quick, easy, and convenient for them. However, there is still a need to conduct further research to explore the factors influencing these behaviors and assess the actual self-medication practices in real-life scenarios.

## Introduction

Self-medication is the practice of accessing and using different drugs for various purposes, including self-diagnosis and self-treatment, without consulting a healthcare professional[1]. The World Health Organization (WHO) defines self-medication as “the use of drugs to treat self-diagnosed diseases or symptoms, or the intermittent or continued use of a prescribed drug for chronic or recurrent disease or symptoms”[2].

**HIGHLIGHTS**

- 76.9% of MBBS students practiced self-medication in the past 6 months.
- Students had strong knowledge, with a mean score of 5.32 ± 0.86.
- Positive attitudes observed, with 69.6% favoring self-medication.
- Findings call for education on safe and responsible drug use.

Self-medication comprises taking without any proper prescription from a physician, using leftover drugs from previous treatments, and obtaining medicines from others[3]. Self-medication includes the selection and use of medicines by individuals to treat self-diagnosed conditions, along with giving medications to household members or close ones without any proper prescription[4].

Even though proper self-medication is encouraged by the WHO for mild disease conditions, it is becoming a global issue with serious repercussions[2]. Despite increasing awareness about the risks of unsupervised drug use, studies continue to show a high prevalence of self-medication among medical undergraduates in Pakistan. This suggests a disconnect between knowledge and safe practice, raising concerns about future prescribing habits and healthcare behavior. Self-medication has been integrated into the healthcare systems of many countries around the world and has several social and economic benefits, but it comes with its associated risks, too[5]. The irresponsible and unmonitored practice of consuming medicines and drugs can lead to various health-related issues, such as delay in proper diagnosis and treatment, exposure to drug-drug interactions, inability to identify contraindications, misdiagnosis, the development of tolerance, serious side effects, dependence, drug abuse, microbial resistance, etc^[^6,7^]^.

There are various factors that are associated with the practice of self-medication, including literacy level, socioeconomic status, easy accessibility to healthcare information and facilities, exposure to drug promotions, awareness about various diseases, and the widespread availability of over-the-counter (OTC) medicines^[^8–11^]^. Although several studies have been conducted on this topic in various regions of Pakistan, there is a lack of recent, institution-specific data that evaluates not only the prevalence but also the nuanced knowledge, attitudes, and reasons for such practices among medical undergraduates. Moreover, most prior studies lacked psychometric validation of the assessment tools or stratified analysis by academic year or gender.

According to some studies, level of education, family history, societal background, and availability of drugs are also considered contributing factors toward the practice of self-medication^[^12,13^]^. High levels of education and professional status are also reported to be factors contributing to self-medication[14]. Since healthcare students, particularly students of medicine, are future health practitioners and physicians, investigating and analyzing their self-medication habits and practices will determine how they will protect themselves and others from improperly utilizing and consuming various medicines and drugs. As students advance in their academic years, their exposure to pharmacology and clinical scenarios increases, influencing their tendency to self-medicate. Senior students reported higher rates of self-medication due to increased confidence in diagnosing minor ailments and familiarity with commonly used drugs.Medical education enhances students’ knowledge of drug indications, side effects, and interactions, contributing to a more informed – though not always safe – approach to self-medication. However, overconfidence may overshadow cautious behavior, leading to inappropriate use in some cases.

The practice of self-medication among medical or health sciences students cannot be denied. Various studies from Saudi Arabia[15], India[16], Uganda[17], Ethiopia^[^18,19^]^, Kuwait[20], and Pakistan^[^3,21–25^]^ reveal a significant prevalence of self-medication by medical or health students. According to the WHO, the purchase of prescription-only drugs without a prescription is much more common than the purchase of OTC drugs and is an accepted practice in developing countries worldwide[26]. Irrational and unreasonable medication practices, including self-medication, pose a serious threat to various professions of medicine and health sciences and have the potential to damage the reputation and image of this sector[25]. Reasonable and safe medication practices can further instill the general public’s confidence in this sector.

Comparing self-medication practices among medical students across Pakistan, a descriptive cross-sectional study conducted online to determine the prevalence of self-medication practices among medical students in Pakistan from 25 January to 20 February 2021 revealed that self-medication was quite prevalent, with 83% of the study population practicing self-medication and the most commonly utilized drugs being antipyretics/analgesics (65.2%) followed by multivitamins (56.0%)[22]. A majority (68%) viewed self-medication as acceptable for minor ailments. Their attitudes significantly influenced health decisions, and they often bypassed formal consultation for conditions they felt confident managing themselves. Easy access to textbooks, online medical databases, and peer knowledge contributes to a high prevalence of self-medication. The lack of structured institutional guidance or counselling on the risks of unsupervised drug use further reinforces this behavior.

Similarly, a descriptive cross-sectional study carried out among medical students from private medical colleges in Pakistan in 2019 found the overall prevalence of self-medication among medical students to be 88.4%, with the most commonly consumed drugs being painkillers (77.57%), followed by antipyretics (52.06%) and cold/flu medicines (48.71%)[25]. Another similar descriptive cross-sectional study conducted among undergraduate students of medical colleges in Pakistan also revealed self-medication to be prevalent among 99% of subjects of the study population, with OTC drugs being most commonly used for self-medication (98.3%)[21].

These studies reveal that self-medication practices are quite common among medical students in Asia, and especially in Pakistan. Self-medication is becoming a serious issue around the world and in Pakistan, which, if not addressed and corrective measures are not taken, can pose some serious risks for the healthcare systems. This study seeks to answer the following research question: *What is the prevalence of self-medication among medical undergraduates, and how are their knowledge, attitudes, and practices (KAP) influenced by demographic and academic factors?* We hypothesize that a significant proportion of students self-medicate, with variations based on academic year and gender. The objective of this study is to assess the prevalence and patterns of self-medication and to analyze the knowledge and attitudes associated with it among medical undergraduates. The findings aim to guide curriculum designers, policymakers, and healthcare institutions in developing targeted interventions. While this study focuses on a specific medical institution in Pakistan, the insights gained may reflect broader trends in South Asian medical education settings. However, the generalizability may be limited, and further multicenter studies are warranted

## Materials and methods

### Study design and rationale

This cross-sectional study was conducted from May 2024 to August 2024 among undergraduate medical students to determine the prevalence, knowledge, attitudes, and practices of self-medication. The strengthening of the reporting of cohort studies in surgery (STROCSS 2024) criteria were followed throughout.

### Eligibility criteria

All undergraduate medical students enrolled during the study period were eligible if they were present on campus, willing to participate, and provided informed consent. Exclusion criteria included students who were absent on the day of data collection, those who refused to participate, and those who submitted incomplete questionnaires.

### Setting and data collection

The questionnaire was administered in person during scheduled class sessions to ensure maximum participation. Students absent during the initial administration were approached in follow-up sessions to preserve the randomization integrity of the sampling method. Participation was voluntary, with no monetary or academic incentives offered.

Data collection was conducted using a printed, paper-based questionnaire distributed by trained study group members under the supervision of the principal author. All data collection processes and completed forms were reviewed and checked by the project director.

### Variables and operational definitions

Self-medication practice: Use of a drug or medication for the treatment of a physical or psychological ailment without a prescriber’s consultation.

Good knowledge: Knowledge score equal to or above the median score (3.0).

Bad knowledge: Knowledge score below the median score (3.0).

Positive attitude: Attitude score above 20. Negative Attitude: Attitude score below 20.

### Bias

Several measures were adopted to reduce bias. Stratified random sampling minimized selection bias by ensuring proportional representation across academic years and gender. Expert review and pilot testing enhanced content validity, while follow-up sessions reduced nonresponse bias. The voluntary, anonymous design reduced social desirability bias.

### Questionnaire development and validation

The questionnaire was adapted from validated instruments assessing knowledge, attitudes, and practices regarding self-medication. Face and content validity were assessed by a panel of three medical education experts, leading to minor modifications for clarity and cultural relevance.

A pilot study involving 40 medical undergraduates (excluded from the final study) yielded a Cronbach’s alpha of 0.78, demonstrating acceptable internal consistency. Unclear or ambiguous items were rephrased or removed. The final tool comprised four sections: demographic information, knowledge-related questions, attitude-related questions, and practice-related questions.

### Sample size determination

Sample size was calculated using the standard single-population proportion formula, assuming a prevalence of 45%, 95% confidence interval, 5% margin of error, and 10% nonresponse rate. The 45% prevalence estimate was chosen due to variability in prior studies from Pakistan, though later evidence, including our findings, suggests a prevalence of ~75%. The conservative estimate was retained to maintain methodological rigor, and this limitation has been acknowledged.

### Sampling procedure

A two-stage stratified random sampling technique was used. First, the student population was stratified by academic year (first to final year), with proportional allocation applied to each stratum. Second, within each year, stratification by gender ensured balanced representation of male and female students. Gender was deliberately included as a stratifying variable, given known differences in healthcare-seeking behaviors and medication use patterns reported in prior literature.

### Data analysis

All data were entered and analyzed using IBM Statistical Package for the Social Sciences (SPSS) Statistics version 25. Descriptive statistics (frequencies, percentages, means, and standard deviations) summarized the data. Chi-square Tests assessed associations between categorical variables such as year of study, gender, and residence with self-medication practices. Subgroup analyses explored variations in knowledge, attitudes, and practices across demographic strata. A *P*-value < 0.05 was considered statistically significant.

### Ethical considerations

The study adhered to the Declaration of Helsinki. Ethical approval was obtained from the Institutional Review Board (IRB/2024-514). Informed consent was obtained from all participants, who were assured of their right to withdraw at any time. Data confidentiality and anonymity were strictly maintained, and all responses were securely stored. Participation was voluntary, and no obligation or coercion was applied.

### Compliance with STROCSS criteria

The study methodology has been designed, conducted, and reported in accordance with the STROCSS guidelines to ensure transparency, reproducibility, and methodological rigor[27].

## Results

Out of the total sample size of 203, 191 students responded to the study, with a response rate of 94%.

**Figure 1.**
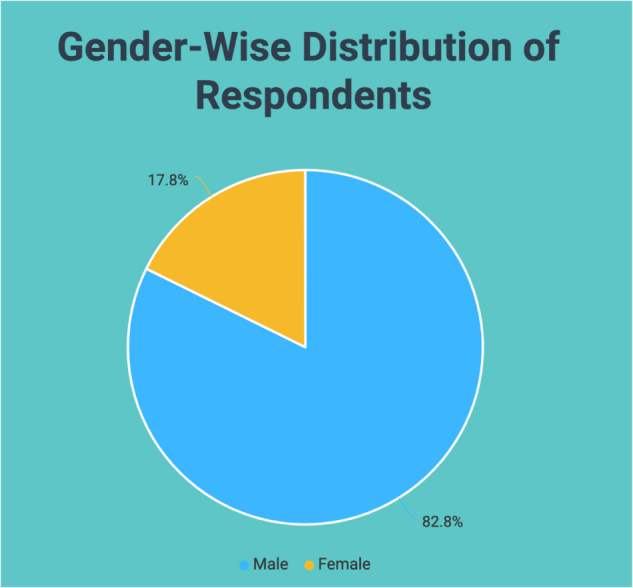
Gender-wise distribution of respondents.

**Figure 2.**
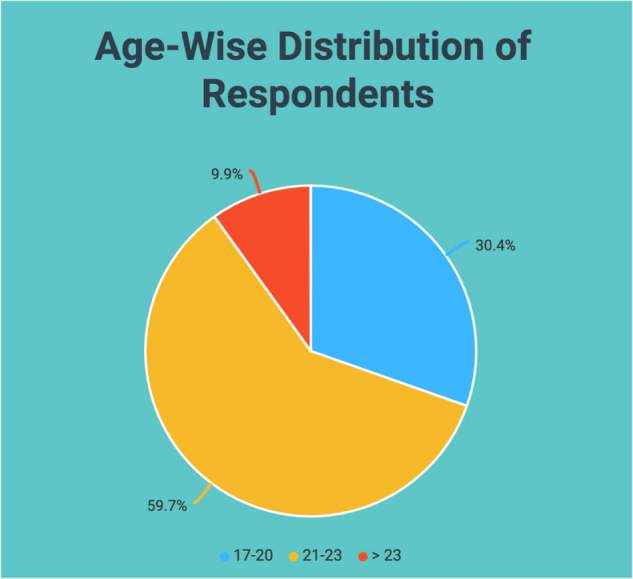
Age-wise distribution of respondents.

As shown in Figure 1, among the respondents, 82.2% were male, and 17.8% were female. Table 1 and Figure 2 illustrate that 59.7% of the respondents belonged to the age group “21–23 years,” 30.4% belonged to the age group “17–20 years,” and 9.9% belonged to the age group “>23 years.” 61.8% resided in urban areas, and 88.55% reported having no known illness.

**Table 1:** Social-demographics characteristics of respondents

Variables	Frequency %					
	1st Year	2nd Year	3rd Year	4th Year	Final Year	Total
Gender						
Male	36 (23.0)	42 (26.8)	39 (24.8)	17 (10.8)	23 (14.6)	157 (82.2)
Female	12 (35.3)	4 (11.8)	9 (26.5)	5 (14.7)	4 (11.7)	34 (17.8)
Age						
17-20	48 (82.8)	10 (17.2)	0	0	0	58 (30.4)
21-23	0	36 (31.6)	45 (39.5)	20 (17.5)	13 (11.4)	114 (59.7)
>23	0	0	3 (15.8)	2 (10.5)	14 (73.7)	19 (9.9)
Residence						
Urban	34 (28.8)	21 (17.8)	33 (28.0)	13 (11.0)	17 (14.4)	118 (61.8)
Rural	14 (19.2)	25 (34.3)	15 (20.5)	9 (12.3)	10 (13.7)	73 (38.2)
Known Illness						
Yes	2 (9.1)	4 (18.2)	11 (50.0)	3 (13.6)	2 (9.1)	22 (11.5)
No	46 (27.2)	42 (24.9)	37 (21.9)	19 (11.2)	25 (14.8)	169 (88.5)

### Students’ knowledge about self-medication

Students’ knowledge about self-medication is presented in Table 2.

**Table 2:** Students’ knowledge about self-medication

Knowledge Related Questions	Frequency %					
	1st Year	2nd Year	3rd Year	4th Year	Final Year	Total
Self-medication is self-consuming medication without the prescriber’s advice						
Yes	46 (26.6)	39 (22.5)	42 (24.3)	21 (12.1)	25 (14.5)	173 (90.6)
No	2 (16.7)	6 (50.0)	4 (33.3)	0	0	12 (6.3)
Don’t Know	0	1 (6.3)	2 (12.5)	1 (6.2)	2 (12.5)	6 (3.1)
Self-medication may not always be safe and effective						
Yes	43 (24.4)	41 (23.3)	46 (26.1)	21 (11.9)	25 (14.2)	176 (92.1)
No	3 (30.0)	3 (30.0)	1 (10.0)	1 (10.0)	2 (20.0)	10 (5.2)
Don’t Know	2 (40.0)	2 (40.0)	1 (20.0)	0	0	5 (2.6)
All medications (prescription, OTC, and herbal) may have adverse effects						
Yes	36 (23.7)	33 (21.7)	40 (26.3)	21 (13.8)	22 (14.5)	152 (79.6)
No	8 (27.6)	11 (37.9)	5 (17.2)	0	5 (17.2)	29 (15.2)
Don’t Know	4 (40.0)	2 (20.0)	3 (30.0)	1 (10.0)	0	10 (5.2)
Increasing or decreasing medication doses without a doctor’s consultation can be dangerous						
Yes	44 (24.3)	41 (22.7)	48 (26.5)	22 (12.1)	26 (14.4)	181 (94.8)
No	3 (60.0)	2 (40.0)	0	0	0	5 (2.6)
Don’t Know	1 (20.0)	3 (60.0)	0	0	1 (20.0)	5 (2.6)
In case of adverse effects, physician’s help must be sought.						
Yes	46 (24.7)	44 (23.7)	47 (25.3)	22 (11.8)	27 (14.5)	186 (97.4)
No	1 (33.3)	2 (66.7)	0	0	0	3 (1.6)
Don’t Know	1 (50.0)	0	1 (50.0)	0	0	2 (1.0)
Self-medication can mask the signs and symptoms of some diseases.						
Yes	29 (19.3)	36 (24.0)	40 (16.7)	21 (14.0)	24 (16.0)	150 (78.5)
No	10 (83.3)	2 (16.7)	0	0	0	12 (6.3)
Don’t Know	9 (31.0)	8 (27.6)	8 (27.6)	1 (3.5)	3 (10.3)	29 (15.2)

The mean knowledge score in this study was determined to be 5.32 ± 0.86. All of the students (191) had satisfactory knowledge about self-medication, with the minimum knowledge score of the respondents being 3, which is equal to or above the median knowledge cumulative score. As summarized in Table 2, 173 (90.6%) agree that self-medication is consuming medication without a prescription. One hundred seventy-six (92.1%) agreed with the statement that self-medication may not always be safe and effective. Only 152 (79.6%) recognized that all drugs, whether prescription, OTC, or herbal, can have adverse effects.

A majority of the students (94.8%) agreed that increasing or decreasing doses on their own could be risky. One hundred eighty-six (97.4%) students believed that the help of a physician must be sought in case of adverse effects, and only 150 (78.5%) students believed that self-medication can mask the signs and symptoms of some medical conditions.

### Students’ attitude towards self-medication

Students’ attitude toward self-medication is presented in Table 3.

**Table 3:** Students’ attitude toward self-medication

Attitude Related Questions	Frequency %					
	1st Year	2nd Year	3rd Year	4th Year	Final Year	Total
Self-medication is part of self-care						
Strong Agree	6 (46.1)	2 (15.4)	2 (15.4)	0	3 (23.1)	13 (6.8)
Agree	4 (14.8)	9 (33.3)	9 (33.3)	1 (3.7)	4 (14.8)	27 (14.1)
Neutral	16 (23.2)	14 (20.3)	20 (29.0)	8 (11.6)	11 (15.9)	69 (36.1)
Disagree	20 (27.4)	18 (24.7)	16 (21.9)	11 (15.0)	8 (11.0)	73 (38.2)
Strongly Disagree	2 (22.2)	3 (33.3)	1 (11.1)	2 (22.2)	1 (11.1)	9 (4.7)
No need for training for self-medication						
Strong Agree	0	2 (28.6)	3 (42.9)	0	2 (28.6)	7 (3.7)
Agree	8 (25.8)	10 (32.3)	5 (16.1)	5 (16.1)	3 (9.7)	31 (16.2)
Neutral	10 (52.6)	2 (10.5)	6 (31.6)	1 (5.3)	0	19 (9.9)
Disagree	25 (25.0)	24 (24.0)	24 (24.0)	11 (11.0)	16 (16.0)	100 (52.4)
Strongly Disagree	5 (14.7)	8 (23.5)	10 (29.4)	5 (14.7)	6 (17.6)	34 (17.8)
Medical students are able to diagnose different diseases						
Strong Agree	2 (28.6)	3 (42.8)	0	0	2 (28.6)	7 (3.7)
Agree	17 (23.6)	15 (20.8)	18 (25.0)	8 (11.1)	14 (19.4)	72 (37.7)
Neutral	17 (25.6)	16 (25.0)	16 (25.0)	7 (10.9)	8 (12.5)	64 (33.5)
Disagree	10 (24.0)	10 (24.0)	13 (31.7)	5 (12.2)	3 (7.3)	41 (21.5)
Strongly Disagree	2 (28.6)	2 (28.6)	1 (14.2)	2 (28.6)	0	7 (3.7)
Medical students are able to treat different diseases						
Strong Agree	1 (25.0)	2 (50.0)	0	0	1 (25.0)	4 (2.1)
Agree	9 (27.3)	5 (15.1)	6 (18.2)	4 (12.1)	9 (27.3)	33 (17.3)
Neutral	15 (23.1)	16 (24.6)	18 (27.7)	8 (12.3)	8 (12.3)	65 (34.0)
Disagree	20 (26.0)	18 (23.4)	22 (28.6)	9 (11.7)	8 (10.3)	77 (40.3)
Strongly Disagree	3 (25.0)	5 (41.7)	2 (16.7)	1 (8.3)	1 (8.3)	12 (6.3)
Do I recommend self-medication to others?						
Strong Agree	6 (85.7)	0	0	1 (14.3)	0	7 (3.7)
Agree	5 (31.3)	4 (25.0)	4 (25.0)	1 (6.2)	2 (12.5)	16 (8.4)
Neutral	11 (25.0)	14 (31.8)	8 (18.2)	7 (15.9)	4 (9.1)	44 (23.0)
Disagree	13 (15.1)	20 (23.3)	28 (32.6)	9 (10.5)	16 (18.6)	86 (45.0)
Strongly Disagree	13 (34.2)	8 (21.0)	8 (21.0)	4 (10.5)	5 (13.2)	38 (19.9)
Easy access to healthcare information and facilities is the main cause that medical students practice self-medication						
Strong Agree	4 (12.5)	7 (21.9)	8 (25.0)	4 (12.5)	9 (28.1)	32 (16.8)
Agree	17 (18.1)	23 (24.5)	27 (28.7)	13 (13.8)	14 (14.9)	94 (49.2)
Neutral	15 (38.5)	10 (25.6)	7 (17.9)	4 (10.3)	3 (7.7)	39 (20.4)
Disagree	11 (50.0)	4 (18.2)	5 (22.7)	1 (4.5)	1 (4.5)	22 (11.5)
Strongly Disagree	1 (25.0)	2 (50.0)	1 (25.0)	0	0	4 (2.1)
The availability of over-the-counter medicines and belief in their safety leads to the use of self-medication						
Strong Agree	8 (20.0)	6 (10.0)	7 (17.5)	11 (27.5)	8 (20.0)	40 (20.9)
Agree	27 (24.8)	26 (23.9)	32 (29.3)	7 (6.4)	17 (15.6)	109 (57.1)
Neutral	7 (26.9)	10 (38.5)	7 (26.9)	1 (3.8)	1 (3.8)	26 (13.6)
Disagree	6 (37.5)	4 (25.0)	2 (12.5)	3 (18.8)	1 (6.2)	16 (8.4)
Strongly Disagree	0	0	0	0	0	0

The mean attitude score of the respondents was determined to be 21.26 ± 3.12. Out of 191 total respondents, 133 (69.6%) students had a good attitude toward self-medication. As shown in Table 3, among the respondents, 13 (6.8%) strongly agreed, and 27 (14.1%) agreed that self-medication is part of self-care, while 73 (38.2%) disagreed and 9 (4.7%) strongly disagreed. Thirty-eight (19.9%) responded positively that there is no need for training for self-medication. Seventy-nine (41.4%) agreed that medical students are able to self-diagnose different diseases, and 37 (19.4%) agreed that medical students are able to self-treat different diseases.

A majority of the respondents (64.9%) disagreed with recommending self-medication to others. Sixty-six percent agreed that easy access to healthcare information and facilities is the main cause of medical students, and 78% believed that the availability of OTC medicines and belief in their safety leads to the use of self-medication.

### Students’ practice of self-medication

The self-medication pattern of the students is presented in Table 4. One hundred forty-seven (76.9%) of the study participants practiced self-medication at least once within the last 6 months. About 149 (41.0%) of them knew that their medications needed a prescription.

**Table 4:** Self-medication practices of students

Self-Medication Practice Evaluating Questions	Frequency %					
	1st Year	2nd Year	3rd Year	4th Year	Final Year	Total
How frequently did you visit the pharmacy for self-medication in the last 6 Months?						
Once	6 (17.2)	7 (20.0)	9 (25.7)	4 (11.4)	9 (25.7)	35 (18.3)
Twice	8 (20.5)	9 (23.1)	13 (33.4)	4 (10.2)	5 (12.8)	39 (20.4)
3 Times	4 (12.5)	12 (37.5)	8 (25)	4 (12.5)	4 (12.5)	32 (16.8)
4 Times	3 (23.1)	4 (30.8)	6 (46.1)	0	0	13 (6.8)
>4 Times	7 (25)	5 (17.9)	5 (17.9)	6 (21.3)	5 (17.9)	28 (14.7)
Never	20 (45.4)	9 (20.4)	7 (16.0)	4 (9.1)	4 (9.1)	44 (23.0)
Do you know your medicines need a prescription?						
Yes	38 (25.5)	35 (23.5)	40 (26.8)	16 (10.8)	20 (13.4)	149 (78.0)
No	10 (23.8)	11 (26.2)	8 (19.1)	6 (14.2)	7 (16.7)	42 (22.0)
Which of the following drugs have you taken for self-medication during the last 6 months?						
Antibiotics	5 (13.5)	12 (32.4)	12 (32.4)	4 (10.8)	4 (10.8)	37 (19.4)
Analgesics (Painkillers)	13 (24.1)	12 (22.2)	13 (24.1)	8 (14.8)	8 (14.8)	54 (28.3)
Anti-Allergics	4 (26.7)	6 (40.0)	2 (13.3)	2 (13.3)	1 (6.7)	15 (7.8)
GIT Drugs	5 (55.5)	0	4 (44.5)	0	0	9 (4.7)
More than one drug	6 (12.0)	9 (18.0)	14 (28.0)	8 (16.0)	13 (26.0)	50 (26.2)
None	15 (57.7)	7 (26.9)	3 (11.5)	0	1 (3.8)	26 (13.6)
The indications for which have you taken self-medications without a prescription during the last 6 months?						
Headache	15 (27.8)	12 (22.2)	9 (16.7)	10 (18.5)	8 (14.8)	54 (28.3)
Fever	9 (39.1)	7 (30.4)	6 (26.1)	0	1 (4.3)	23 (12.0)
Cough	2 (12.5)	6 (37.5)	4 (25.0)	4 (25.0)	0	16 (8.4)
Diarhhea	6 (60.0)	1 (10.0)	1 (10.0)	1 (10.0)	1 (10.0)	10 (5.2)
Allergy	3 (23.1)	4 (30.7)	3 (23.1)	0	3 (23.1)	13 (6.8)
More than one indication	12 (18.7)	13 (20.3)	19 (29.7)	7 (10.9)	13 (20.3)	64 (33.5)
None	1 (9.1)	3 (27.3)	6 (54.5)	0	1 (9.1)	11 (5.8)
What is your source of information for self-medication?						
Healthcare Professionals	17 (28.8)	19 (32.2)	12 (20.3)	5 (8.5)	6 (10.2)	59 (30.9)
Friends	7 (33.3)	6 (28.6)	7 (33.3)	1 (4.8)	0	21 (11.0)
Internet	11 (25.6)	11 (25.6)	11 (25.6)	3 (6.8)	7 (16.3)	43 (22.5)
Books	3 (11.5)	2 (7.7)	4 (15.4)	9 (34.6)	8 (30.8)	26 (13.6)
Self	4 (16.7)	3 (12.5)	8 (33.3)	3 (12.5)	6 (25.0)	24 (12.6)
Others	6 (33.3)	5 (27.8)	6 (33.3)	1 (5.5)	0	18 (9.4)
From where do you buy self-medication drugs?						
Pharmacy	40 (23.2)	43 (25.0)	44 (25.6)	20 (11.6)	25 (14.5)	172 (90.0)
Herbal Pharmacy	1 (33.3)	0	1 (33.3)	1 (33.3)	0	3 (1.6)
Relative	6 (66.7)	1 (11.1)	2 (22.2)	0	0	9 (4.7)
Others	1 (14.2)	2 (28.6)	1 (14.3)	1 (14.3)	2 (28.6)	7 (3.7)
Why do you prefer to use self-medication?						
Convenience	7 (21.9)	7 (21.9)	5 (15.6)	4 (12.5)	9 (28.1)	32 (16.7)
Cost-effectiveness	7 (50.0)	3 (21.4)	3 (21.4)	1 (7.1)	0	14 (7.4)
Familiarity	11 (31.4)	7 (20.0)	9 (25.7)	3 (8.6)	5 (14.3)	35 (18.3)
Timeliness	16 (19.7)	23 (28.4)	22 (27.2)	12 (14.8)	8 (9.9)	81 (42.4)
Previous Successful Outcomes	2 (25.0)	0	2 (25.0)	1 (12.5)	3 (37.5)	8 (4.2)
Others	5 (23.8)	6 (28.6)	7 (33.3)	1 (4.8)	2 (9.5)	21 (11.0)
Have you ever experienced adverse effects after self-medication?						
Yes	12 (26.7)	13 (28.9)	8 (17.8)	2 (4.4)	10 (22.2)	45 (23.6)
No	36 (24.6)	33 (22.6)	40 (27.4)	20 (13.7)	17 (11.6)	146 (76.4)
Do you feel confident with self-medication?						
Yes	25 (23.6)	24 (22.6)	26 (24.5)	15 (14.1)	16 (15.1)	106 (55.5)
No	23 (27.1)	22 (25.9)	22 (25.9)	7 (8.2)	11 (12.9)	85 (44.5)

**Figure 5.**
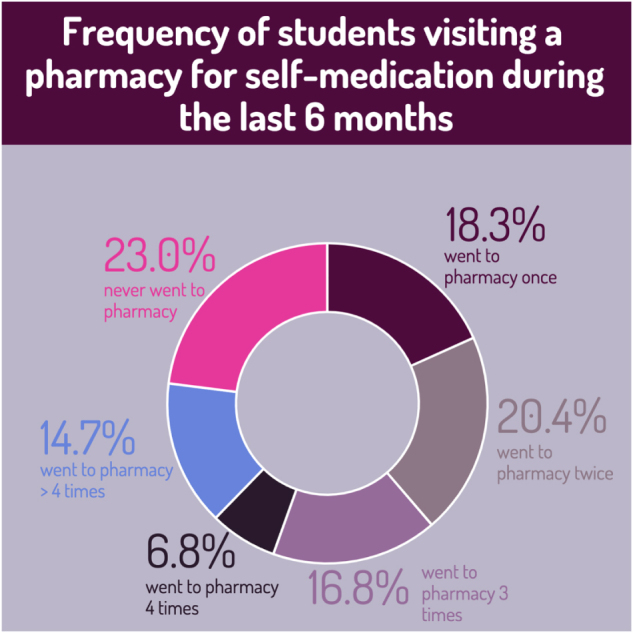
Percentage of most common indications for which students self-medicated during the past 6 months.

The most commonly self-consumed drugs, as shown in Figure 4, were analgesics (28.3%), followed by self-consumption of more than one drug (26.2%) during the last 6 months. 33.5% of the participants self-treated for more than one indication, followed by 28.3% for headache and 12.0% for fever shown in Figure 5, during the last 6 months.

**Figure 3.**
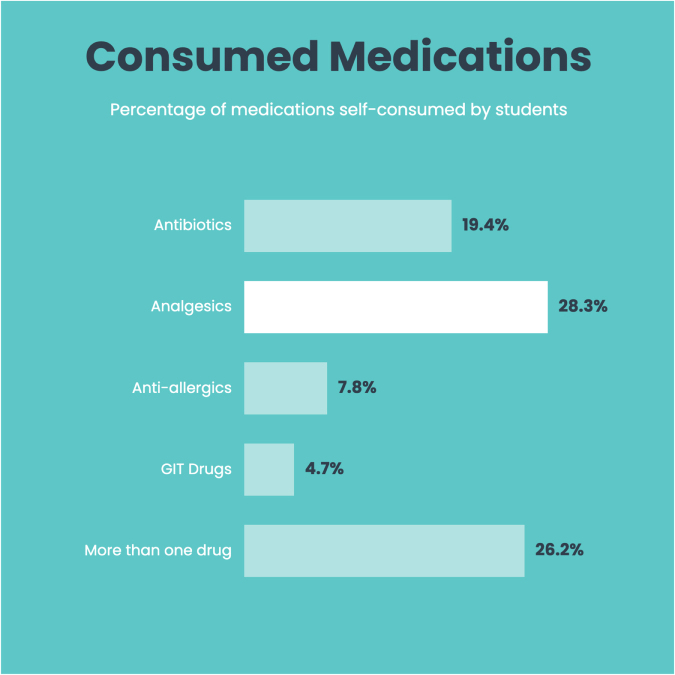
Frequency of students visiting a pharmacy for self-medication during the last 6 months.

As illustrated in Figure 6, 30.9% of the participants considered healthcare professionals their primary source of information for self-medication, followed by the internet (22.5%) and books (13.6%). A majority of the participants (90%) obtained drugs for self-medication from pharmacies, as shown in Figure 3, while only a few obtained them from other sources: herbal pharmacies (1.6%) and relatives (4.7%).

**Figure 4.**
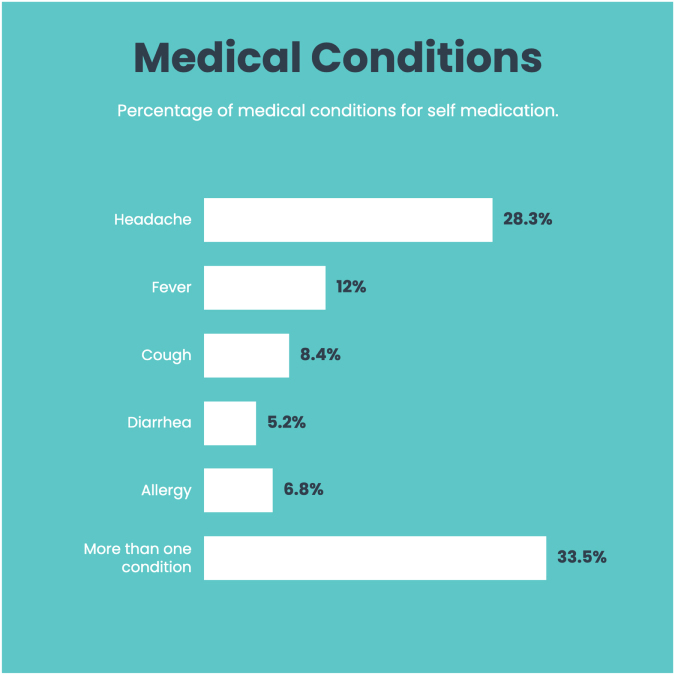
Percentage of most commonly self-consumed medications by students during the past 6 months.

**Figure 6.**
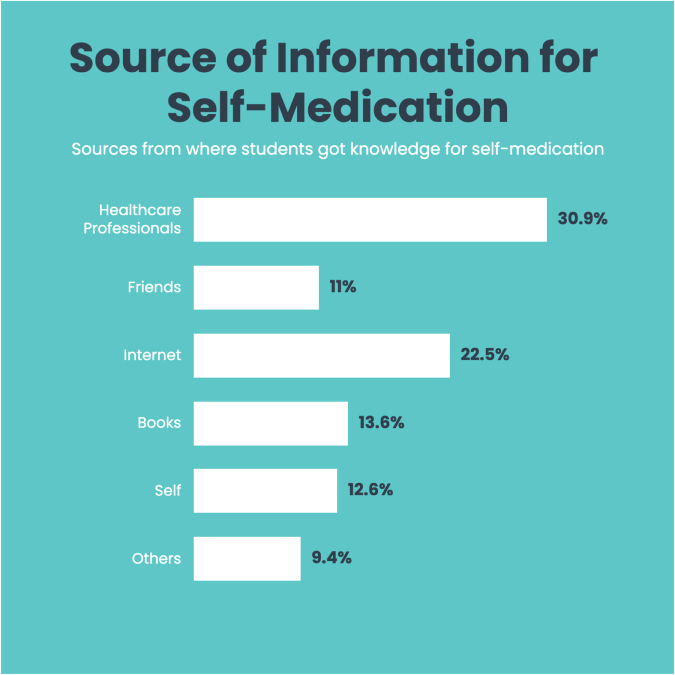
Sources from where students obtained knowledge for self-medication.

**Figure 7.**
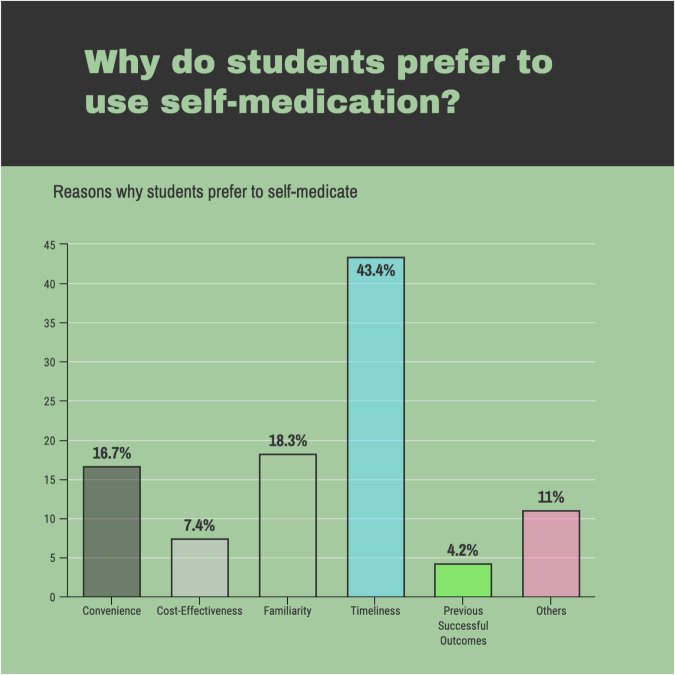
Reasons why students prefer to self-medicate.

The most common reason for practicing self-medication, as described in Figure 7, was timeliness (42.4%), followed by familiarity (18.3%) and convenience (16.7%). The majority of the respondents (76.4%) did not experience any adverse effects related to their medications. More than half of the students (5.5%) felt confident while using self-medication as part of their healthcare.

## Discussion

### Self-medication prevalence

The present study revealed that the prevalence of self-medication among medical students was 76.9%. This is a very significant number, which is almost consistent with similar study reports from universities (72.7%)[6], (63.9%)[15], (64.98%)[18] and Indian colleges (74.6 and 69.4%)[28]. But the prevalence is considerably higher than similar study reports of the university in Bahrain (44.8%)[20], universities in Kuwait (35.9%)[29], Iran (57.1%)[30], and students and other population groups in the developed western regions such as German (8%)[31], Spain (45%)[31], Italy (53.4%)[32], and Norway (54%)[33].

Still, there are some studies indicating a higher prevalence of self-medication among health science students in Europe and other developed countries such as Serbia (81.3%)[34], Slovenia (92.3%)[35], and Australia (91.7%)[36]. From these reports, it can be said that self-medication is a commonly practiced part of healthcare all over the world, in varying degrees. Generally, self-medication prevalence is reported to be higher in developing than in developed countries[37]. The welfare status, income per capita, better quality healthcare, and a more efficient drug supply management system can be listed as the main factors. In addition, educational level and specialty, socioeconomic differences, acquired knowledge about specific disease perceptions, and other related sociodemographic variations could be reasons for the similarities and differences among those reports.

### Knowledge about self-medication

Self-medication has become a global habit nowadays, where individuals in every country of the world start to self-medicate just based on symptom perception[38]. Although the WHO encourages self-medication, the consequences of self-medication have shown that self-medication is becoming a global health issue[39]. For this reason, health science students, especially Medical students, are expected to have good knowledge regarding the appropriate use of drugs and medicines. This study revealed that all of the students in the study (100%) had satisfactory knowledge about self-medication. This is in contrast to the overall knowledge score results in different countries of the world, including India (55%)[40], Taiwan (45.8%)[41], Oman (75%)[42], Kuwait (53.5%)[20], and Iran (16%)[30]. These differences could be due to multiple factors, including the level of education, the year of study of respondents, the level and quality of academic institutions, and the socio-demographic backgrounds of the respondents.

Based on the results of this study, about 173 (90.6%) of respondents agreed that self-medication is consuming medication without a prescription, and 152 (79.6%) recognized that all drugs, whether prescription, OTC, or herbal, can have adverse effects. A majority of the students (94.8%) agreed that increasing or decreasing doses on their own could be risky. One hundred eighty-six (97.4%) students believed that the help of a physician must be sought in case of adverse effects, and only 150 (78.5%) students believed that self-medication can mask the signs and symptoms of some medical conditions. While a study conducted at a university in Saudi Arabia showed that about 81.6% agree that self-medication is consuming medication without a prescription, 94.3% of the participants recognized that all drugs can have adverse effects, 97.2% agreed that increasing or decreasing doses on their own can be dangerous, and 94.9% of the participants believed that in case of adverse effect, physician help must be sought, while 88.3% of participants believed that self-medication can mask the signs and symptoms of some medical condition[15].

A study conducted at a medical university in Pakistan in 2019 revealed that 46.3% of the participants had knowledge of the dosage of drugs and 41.5% had knowledge about precautions for their use[43]. The difference in the values of these studies suggests

1. A potential gap in medical education and the content and structure of medical curricula can significantly impact students’ knowledge of self-medication.
2. Differences in cultural and regional influences, cultural norms, and regional healthcare practices can shape students’ perspectives on self-medication.
3. Healthcare system accessibility and the availability of services may impact self-medication knowledge.
4. Awareness of risks and benefits.

Acknowledging these influences is crucial for designing targeted interventions aimed at enhancing responsible self-medication practices.

To enrich the interpretive depth of this study, the discussion has been broadened to incorporate contextual comparisons with both national and international findings on self-medication practices. This includes analysis of cultural norms, socioeconomic factors, and educational disparities that may influence behavior across different regions. Additionally, the role of media exposure, ease of online access to medical information, and clinical overconfidence, particularly among senior medical students, have been considered as critical determinants. The discussion further highlights curriculum gaps that may contribute to unsafe medication practices despite satisfactory knowledge levels. These insights underline the importance of integrating structured guidance on responsible self-medication into medical education. Ultimately, this expanded scope aims to inform educational policy reforms and encourage safer healthcare behaviors among future professionals.

### Attitude towards self-medication

The positive attitude towards self-medication among 69.6% of students in this study is comparable to the 67% reported in an Indian study[16]. However, it is lower than the 87% observed among Serbian students[34]. This indicates that while a majority of medical students have a favorable self-medication attitude, there is variability across regions, likely due to cultural influences.

Like this study, Sundararajan and Thangappan (2018)[16] also found that only a small proportion of students strongly agreed that self-medication can replace physician consultation. Similarly, a study in Bahrain reported 78% disagreement with self-diagnosis and treatment[20]. This highlights that despite an overall positive attitude, students understand the limitations of self-medication.

Easy access to information and medicines, leading to self-medication, has also been reported by studies in India and Serbia^[^16,34^]^. This suggests that while availability enables self-care, it also increases misuse risks, requiring mitigation through education.

This study provides insights into self-medication attitudes among medical students in Pakistan. The findings are mostly consistent with previous Asian and European studies. However, qualitative research could aid in understanding the reasoning and motivations behind student attitudes. Longitudinal studies are also needed to determine variations across educational levels. The curriculum should emphasize responsible self-medication practices to translate positive attitudes into prudent behaviors.

### The pattern of self-medication practice

According to our study, the most frequently used drugs for self-medication were analgesics (28.3%), followed by self-consumption of more than one drug (26.2%). 33.5% of the participants were self-treated for more than one condition, followed by headache (28.3%) and fever (12.0%). A similar study conducted in India[44] showed that oral antibacterial agents were the most commonly used drugs for self-medication, followed by oral anti-inflammatory agents and antipyretics. Another study conducted in West Bengal[13] also confirmed the practice of self-medication, wherein the most common drugs used were antibiotics (31.09%), followed by analgesics (23.21%), whereas the most common diseases treated were cough and common cold (35.21%), followed by diarrhea (25.47%) and fever (15.73%). A study among medical students in Kathmandu[45] was conducted in which the commonly self-prescribed drugs were analgesics and antipyretics, and headache was the most common disease treated by self-medication. A study conducted among health science students in Ethiopian institutions[46] showed that antipyretics/analgesics were the common drugs and headache was the prominent ailment, whereas results[47] confirmed the use of antibiotics (59.90%) as the most common self-prescribed drugs, followed by analgesics (47.80%) and the common illnesses perceived were headache (46.50%) followed by gastrointestinal diseases (34.10%) and respiratory tract infections (31.80%). NSAIDs (52.7%), antipyretics (13.7%), and antacids (12.7%) were the commonly self-prescribed drugs among students of Saudi Arabia[48], and headache (65.9%), cold/flu (31.2%), and fever (30.2%) were the commonly self-treated ailments. OTC drugs (98.3%) were the most common drugs in practice for self-medication among medical students in Pakistan[49]. A study from university students in Pakistan[49] showed analgesics (88.3%), followed by antipyretics (65.1%) and antibiotics (35.2%), as the most common drugs, and headache (72.4%), followed by flu (65.5%) and fever (55.2%), as the most common symptoms for which self-medication was practiced.

30.9% of the participants considered healthcare professionals as their primary source of information for self-medication, followed by the internet (22.5%) and books (13.6%). A study from Pakistan[49] showed that the media was found to be the most common source of information. Studies from other institutions showed that self-information was the source of information for self-medication practice^[^50,51^]^. The majority of the medical science students in Iran[30] also reported that they select their medicines on their own decision (77.7%). The reason that they rely on self-information for self-medication practice is familiarity with diseases and knowledge about drugs, which gives them enough confidence to treat themselves without consulting a professional.

In our study, the majority of the participants (90%) obtained drugs for self-medication from pharmacies, while only a few of them obtained them from other sources: herbal pharmacies (1.6%) and relatives (4.7%). Reports from other study areas in Ethiopia[52] and Eritrea[53] indicated that drug retailers were the most common source of obtaining drugs for self-medication. Lack of knowledge about proper dosage, drug interactions, and proper dispensing of medications among drug retailers, which, if not taken into account timely, can lead to potential health hazards and serious adverse effects. Other sources, like herbal pharmacies, can also lead to medical malpractice. Relatives from a healthcare professional background may also be a source of access to medications.

Educational strategies that rely solely on lectures or printed materials have shown minimal impact on changing practitioner behavior. In contrast, interactive and multifaceted educational methods such as academic detailing, audit and feedback, and peer-led sessions consistently demonstrate more substantial improvements in clinical decision making[54]. Adapting these evidence-based teaching strategies within the undergraduate medical curriculum through mechanisms like small-group workshops, simulated patient encounters, clinical rotations with pharmacist-led debriefs, and digital reminders could support safer self-medication practices.

Furthermore, curriculum development initiatives in medical entomology have shown the effectiveness of hands-on, problem-based, and student-centered learning approaches in enhancing knowledge retention and clinical relevance[55]. A similar educational approach, emphasizing practical application through case-based learning and integrative modules on pharmacology and rational drug use, may address current gaps and reduce unsafe self-medication behaviors among medical undergraduates.

Based on our study, the most common reason for practicing self-medication was timeliness (42.4%), followed by familiarity (18.3%), and convenience (16.7%). The need for time-saving was the most frequently mentioned reason for self-medication in health science students in Bahrain[29]. Other similar studies in Ethiopia[46] concluded that past experience of the disease and lack of affordability were reported as the major reasons for self-medication. Similar results were reported from research conducted at Asmara College of Health Sciences, Eritrea[53]. The reason for the self-medication practice by the students of Uganda[17] was classifying illnesses as minor (33%), time-saving (15%), having old prescriptions (11%), and high consultation fees (9%). However, treating diseases by oneself based on previous experiences and in order to save time may lead to serious health problems and ultimately major health hazards that may cost a person his/her life.

According to our research, the majority of the respondents (76.4%) did not experience any adverse effects related to their medications. More than half of the students (5.5%) felt confident while using self-medication as part of their healthcare. The study in Eritrea[53] reported adverse reactions among 9.2% of the participants. Thirty-three percent of the participants of a study carried out in South India[44] were unaware of any adverse effects, and 5% did experience adverse effects.

Beyond identifying prevalence and patterns, future studies should explore behavioral determinants of self-medication among medical students, such as academic stress, peer influence, and digital health information use. Qualitative research using focus groups or in-depth interviews could provide a deeper understanding of the psychosocial motivations behind self-medication. Furthermore, longitudinal studies following students across different years of training may clarify whether knowledge and attitudes translate into safer practices over time.

## Conclusion

From this study, it is evident that there is a significantly high prevalence of self-medication practice among medical students. The proportion of students with good knowledge and a positive attitude towards self-medication is high. About 70% of the respondents exhibited a good attitude towards self-medication, showcasing a favorable inclination towards taking responsibility for their own health and well-being. Additionally, it is noteworthy that 100% of the participants demonstrated good knowledge about self-medication, suggesting a high level of awareness and understanding regarding the responsible use of self-medication. Most students prefer self-medication because it is quick, easy, and convenient for them.

This implies that most students are well aware of self-medication and its consequences. Such positive attitudes and knowledge are crucial in promoting safe and effective self-care strategies, especially among future healthcare professionals. However, while most respondents displayed positive attitudes and knowledge, further research may be necessary to explore the factors influencing these behaviors and assess the actual self-medication practices in real-life scenarios.

The limitations of this study include: This study was a “single-center study,” and the results obtained may not be generalizable; the sample size of the study was not adequate; and the study did not take into consideration the cultural variations and financial conditions of the students. In light of the study findings, several implications emerge. Firstly, the results underscore the need for integrating formal education on rational drug use and the risks of self-medication into undergraduate medical curricula. Institutional awareness campaigns should also be implemented to promote responsible self-care and discourage inappropriate self-medication practices. Furthermore, this study highlights the importance of conducting future multicenter and longitudinal research to assess self-medication behaviors across varied academic settings and over time. These insights will aid in formulating comprehensive strategies to address this growing concern among future healthcare providers. Lastly, the conclusion acknowledges key limitations of the study, such as its single-center design and limited generalizability, while also emphasizing its strengths, including a high response rate, a validated data collection instrument, and use of stratified random sampling to ensure representativeness.

### Future research directions

Beyond the current findings, future research should focus on identifying the behavioral and contextual determinants of self-medication among medical students, including factors such as academic stress, peer influence, and the increasing reliance on online health information. Qualitative approaches, such as focus group discussions and in-depth interviews, could provide valuable insights into the psychosocial drivers behind these practices. Longitudinal and multi-center studies are also warranted to assess how knowledge and attitudes evolve throughout medical training and whether they translate into safe and rational self-medication practices.

### Policy implications

From a policy perspective, integrating structured modules on rational drug use and the risks of inappropriate self-medication into undergraduate medical curricula is essential. Institutional awareness campaigns, pharmacist-led workshops, and interprofessional teaching initiatives may further strengthen responsible practices. At a broader level, enforcing stricter regulations on non-prescription drug sales and promoting safe-use guidelines through digital and community-based platforms could help mitigate unsafe self-medication behaviors among future healthcare providers.

## Limitations

While this study provides valuable insights into the knowledge, attitudes, and practices of self-medication among medical undergraduates, subgroup analyses and inferential statistics were not conducted in the initial analysis. This decision was made due to the primary objective of the study being descriptive in nature, focusing on estimating the overall prevalence and patterns of self-medication. Additionally, the limited sample size per subgroup (e.g., gender, academic year, and residence) may have restricted the statistical power required to detect meaningful associations. Future studies with larger and more diverse samples are recommended to explore these subgroup differences more robustly using multivariate techniques.

This study acknowledges potential sources of bias inherent in its design. Selection bias may have occurred, as students who engage in self-medication or possess heightened health awareness may have been more motivated to participate in the survey. Additionally, the reliance on self-reported data introduces the possibility of social desirability bias, wherein participants may have underreported unsafe or excessive self-medication practices to align with socially acceptable norms. These factors may influence the accuracy and generalizability of the findings.

## Data Availability

Any datasets generated during and/or analyzed during the current study are available upon reasonable request

## Research registration unique identifying number (UIN)

Not applicable for this study as it does not involve patients.
